# MASPECTRAS: a platform for management and analysis of proteomics LC-MS/MS data

**DOI:** 10.1186/1471-2105-8-197

**Published:** 2007-06-13

**Authors:** Jürgen Hartler, Gerhard G Thallinger, Gernot Stocker, Alexander Sturn, Thomas R Burkard, Erik Körner, Robert Rader, Andreas Schmidt, Karl Mechtler, Zlatko Trajanoski

**Affiliations:** 1 Institute for Genomics and Bioinformatics and Christian-Doppler Laboratory for Genomics and Bioinformatics, Graz University of Technology, Petersgasse 14, 8010 Graz, Austria; 2 Austrian Research Centers GmbH -ARC, eHealth Systems, Reininghausstrasse 13/1, 8020 Graz, Austria; 3 FH Joanneum, Kapfenberg, Werk-VI-Sraße 46, 8605 Kapfenberg, Austria; 4 Christian Doppler Laboratory for Proteome Analysis, Dr. Bohr-Gasse 3, 1030 Vienna, Austria; 5 Research Institute of Molecular Pathology, Dr. Bohr-Gasse 7, 1030 Vienna, Austria

## Abstract

**Background:**

The advancements of proteomics technologies have led to a rapid increase in the number, size and rate at which datasets are generated. Managing and extracting valuable information from such datasets requires the use of data management platforms and computational approaches.

**Results:**

We have developed the MAss SPECTRometry Analysis System (MASPECTRAS), a platform for management and analysis of proteomics LC-MS/MS data. MASPECTRAS is based on the Proteome Experimental Data Repository (PEDRo) relational database schema and follows the guidelines of the Proteomics Standards Initiative (PSI). Analysis modules include: 1) import and parsing of the results from the search engines SEQUEST, Mascot, Spectrum Mill, X! Tandem, and OMSSA; 2) peptide validation, 3) clustering of proteins based on Markov Clustering and multiple alignments; and 4) quantification using the Automated Statistical Analysis of Protein Abundance Ratios algorithm (ASAPRatio). The system provides customizable data retrieval and visualization tools, as well as export to PRoteomics IDEntifications public repository (PRIDE). MASPECTRAS is freely available at

**Conclusion:**

Given the unique features and the flexibility due to the use of standard software technology, our platform represents significant advance and could be of great interest to the proteomics community.

## Background

The advancement of genomic technologies – including microarray, proteomic and metabolic approaches – have led to a rapid increase in the number, size and rate at which genomic datasets are generated. Managing and extracting valuable information from such datasets requires the use of data management platforms and computational approaches. In contrast to genome sequencing projects, there is a need to store much more complex ancillary data than would be necessary for genome sequences. Particularly the need to clearly describe an experiment and report the variables necessary for data analysis became a new challenge for the laboratories. Furthermore, the vast quantity of data associated with a single experiment can become problematic at the point of publishing and disseminating results. Fortunately, the communities have recognized and tackled the problem through the development of standards for the capturing and sharing of experimental data. The microarray community arranged to define the critical information necessary to effectively analyze a microarray experiment and defined the Minimal Information About a Microarray Experiment (MIAME) standard [1]. Subsequently, MIAME was adopted by scientific journals as a prerequisite for publications and several software platforms supporting MIAME were developed [2,3].

The principles underlying MIAME have reasoned beyond the microarray community. The Proteomics Standards Initiative (PSI) [4] aims to define standards for data representation in proteomics analogues to that of MIAME and developed the Minimum Information About a Proteomics Experiment (MIAPE) standard [5]. An implementation independent approach for defining the data structure of a proteomics experiment, the Proteome Experimental Data Repository (PEDRo) [6] was developed, and a PSI compliant public repository was set up [7]. Hence, given the defined standards and available public repositories, computational systems can now be developed to support proteomics laboratories and enhance data dissemination.

To meet the needs for high-throughput MS laboratories several tools and platforms covering various parts of the analytical pipeline were recently developed including the Trans Proteomics Pipeline [8], The Global Proteome Machine [9], VEMS [10,11], CPAS [12], CHOMPER [13], ProDB [14], PROTEIOS [15], GAPP [16], PeptideAtlas [17], EPIR [18], STEM [19], and TOPP [20] (see additional file 1 for a comparison of the features). However, to the best of our knowledge there is currently no academic or commercial data management platform supporting MIAPE and enabling PRoteomics IDEntifications database (PRIDE) export. Moreover, it became evident that several search engines should be used to validate proteomics results [21]. Hence, a system enabling comparison of the results generated by the different search engines would be of great benefit. Additionally, integration of algorithms for peptide validation, protein clustering and protein quantification into a single analytical pipeline would considerably facilitate analyses of the experimental data.

We have therefore developed the MAss SPECTRometry Analysis System (MASPECTRAS), a web-based platform for management and analysis of proteomics liquid chromatography tandem mass spectrometry (LC-MS/MS) data supporting MIAPE. MASPECTRAS was developed using state-of-the-art software technology and enables data import from five common search engines. Analytical modules are provided along with visualization tools and PRIDE export as well as a module for distributing intensive calculations to a computing cluster.

## Implementation

The application is based on a three-tier architecture, which is separated into presentation-, middle-, and database layer. Each tier can run on an individual machine without affecting the other tiers. This makes every component easily exchangeable. A relational database (MySQL, PostgreSQL or Oracle) forms the database layer. MASPECTRAS follows and extends the PEDRo database schema [6] (see additional file 2) to suit the guidelines of PSI [4]. The business layer consists of a Java 2 Enterprise Edition (J2EE) compliant application which is deployed to the open source application server JBoss [22]. Access to the data is provided by a user-friendly web-interface using Java Servlets and Java Server Pages [23] via the Struts framework [24]. Computational or disk space intensive tasks can be distributed to a separate server or to a computing cluster by using the in-house developed JClusterService interface. This web service based programming interface uses the Simple Object Access Protocol (SOAP) [25] to transfer data for the task execution between calculation server and MASPECTRAS server. The tasks can be executed on dedicated computation nodes and therefore do not slow down the MASPECTRAS web interface. This remote process execution system is used as a backend for the protein grouping analysis, for the mass quantification and for the management of the sequence databases and their sequence retrieval during import.

The current implementation of MASPECTRAS allows the comparison of search results from SEQUEST [26], Mascot [27], Spectrum Mill [28], X! Tandem [29], and OMSSA [30]. The following file formats are supported: SEQUEST: ZIP-compressed file of the *.dta, *.out and SEQUEST.params files; Mascot: *.dat; Spectrum Mill: ZIP-compressed file of the results folder including all subfolders; X! Tandem: the generated *.xml; OMSSA: the generated *.xml with included spectra and search params; Raw data: XCalibur raw format (*.raw) version 1.3, mzXML [31] and mzData [32] format. The data can be imported into MASPECTRAS database asynchronously in batch mode, without interfering with the analysis of already uploaded data. The spectrum viewer applet and the diagrams are implemented with the aid of JFreeChart [33] and Cewolf [34] graphics programming frameworks. The whole system is secured by a user management system which has the ability to manage the access rights for projects and offers data sharing and multiple user access roles in a multi-user environment [2].

## Results

### Analysis pipeline

**Figure 1:**
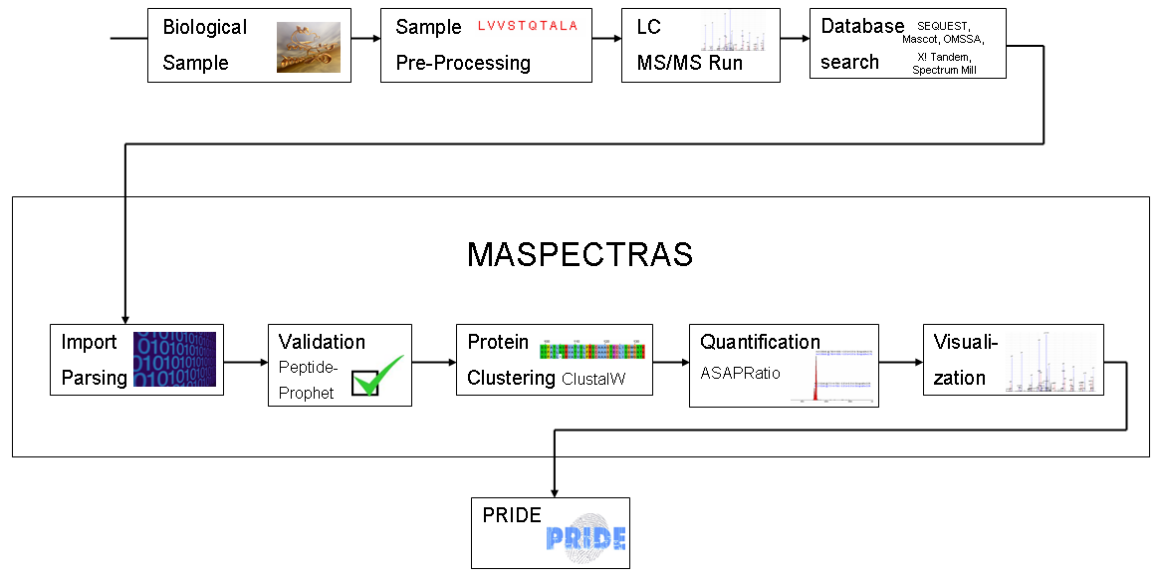
**Schematic overview of the analysis pipeline of MASPECTRAS**. Search results from SEQUEST, Mascot, Spectrum Mill, X! Tandem, and OMSSA are imported and parsed. In the next steps peptides are validated using PeptideProphet [37] and the corresponding proteins clustered based on Markov clustering using BLAST [38], the sequences are aligned with CLUSTAL W [39]. Then the peptides are quantified using the ASAPRatio algorithm [41], the results stored in the database and exported to the public repository PRIDE [7].

MASPECTRAS extends the PEDRo relational database schema and follows the guidelines of the PSI. It accepts the native file formats from SEQUEST [26], Mascot [27], Spectrum Mill [28], X!Tandem [29], and OMSSA [30]. The core of MASPECTRAS is formed by the MASPECTRAS analysis platform (Figure 1). The platform encompasses modules for the import and parsing data generated by the above mentioned search engines, peptide validation, protein clustering, protein quantification, and a set of visualization tools for post-processing and verification of the data, as well as PRIDE export.

#### Import and parsing data from search engines

There are several commercial and academic search engines for proteomics data. Based on known protein sequences stored in a database, these search engines perform *in silico *protein digestion to calculate theoretical spectra for the resulting peptides and compare them to the obtained ones. Based on the similarity of the two spectra, a probability score is assigned. The results (score, peptide sequence, etc.) are stored in a single or in multiple files, and often only an identification string for the protein is stored whereas the original sequence is discarded. However, the search engines are storing different identification strings for the proteins (e.g. X! Tandem: gi|231300|pdb|8GPB|; Spectrum Mill: 231300). Moreover, several databases are not using common identifiers (e.g. National Center for Biotechnology Information non redundant (NCBI nr): gi|6323680; Mass Spectrometry protein sequence DataBase (MSDB) [35]: S39004). In order to compare the search results from different search engines additional information from the corresponding sequence databases is needed. The format of the accession string has to be known to retrieve the protein sequence and additional required information from the sequence database, like protein description, or the organism the protein belongs to. The only common basis within the different databases used by the search algorithms is the amino acid sequence of the proteins. In order to make results of different algorithms comparable and to find the corresponding proteins in the different result files the sequence information is taken as unique identification criterion.

We have developed parsers for the widely used search engines SEQUEST, Mascot, Spectrum Mill, X!Tandem, and OMSSA. MASPECTRAS manages the sequence databases used while searching with different modules internally. Any database available in FASTA format [36] can be uploaded to MASPECTRAS. Parsing rules are user definable and therefore easily adaptable to different types of sequence databases. When results of a search engine are imported into MASPECTRAS, the system first tries to determine whether the same accession string for the same database version exists. If that is not the case, the original sequence information is retrieved from the corresponding sequence database. Subsequently the system tries to match the sequence against the sequences already stored in the database. If an entry with the same sequence information but a different accession string is found, the new accession string is associated with the unique identifier of the already stored sequence. Otherwise a new unique identifier is created and the sequence is stored with the appropriate accession strings.

#### Peptide validation

MASPECTRAS calculates a probability score for SEQUEST and Mascot which is based on the algorithm of PeptideProphet [37]. Data re-scoring adds a further layer, which improves the specificity of the highly sensitive SEQUEST and Mascot database searches. This procedure could be applied to other database search algorithms as well and can additionally offer a remap of the results from different database search algorithms onto one single probability scale. The statistical model incorporates a linear discriminant score based on the database search scores (for SEQUEST: XCorr, dCn, Sp rank, and mass difference) as well as the tryptic termini and missed cleavages [37]. After scoring the data has to pass a user definable filter, which uses the search programs specific score to discard the most unlikely data.

#### Protein clustering

In peptide fragmentation fingerprinting (PFF) peptides are identified by search engines, which have to be mapped to proteins. A single peptide often corresponds to a group of proteins. Therefore, PFF identifies protein groups, each protein sharing similar peptides. A grouped protein view represents the result more concisely and proteins with a small number of identified peptides can be recognized easier in complex samples. The protein grouping implemented in MASPECTRAS is based on Markov clustering [38] using Basic Local Alignment Search Tool (BLAST) and multiple alignments [39]. A file in FASTA format is assembled containing all sequences to be clustered. Each sequence is then compared against each other. The all-against-all sequence similarities generated by this analysis are parsed and stored in an upper triangular matrix. This matrix represents sequence similarities as a connection graph. Nodes of the graph represent proteins, and edges represent sequence similarity that connects such proteins. A weight is assigned to each edge by taking the average pair wise -log_10 _of the BLAST E-value. These weights are transformed into probabilities associated with a transition from one protein to another within this graph. This matrix is passed through iterative rounds of matrix multiplication and inflation until there is little or no net change in the matrix [38]. The final matrix is then interpreted as the protein clustering and the number of the corresponding cluster is stored for every protein hit. The proteins within a group are aligned by CLUSTAL W [39] and visualized by the integrated Jalview Alignment Editor [40]. For proteins with the same sequence from different searches the corresponding protein groups are combined at the time the searches are compared.

#### Protein quantification

For quantification of peptides the ASAPRatio algorithm described in [41] has been integrated and applied. To determine peak area a single ion chromatogram is reconstructed for a given m/z range by summation of ion intensities. This chromatogram is then smoothed tenfold by repeated application of the Savitzky – Golay smooth filtering method [42]. For each isotopic peak center and width are determined. The peak width is primarily calculated by using the standard ASAPRatio algorithm and for further peak evaluation a new algorithm for recognizing peaks with saddlepoints has been implemented. With this algorithm a valley (a local minimum of the smoothed signal) is recognized to be part of the peak and added to the area. The calculated peak area is determined as the average of the smoothed and the unsmoothed peak. From this value background noise is subtracted, which is estimated from the average signal amplitude of the peak's neighborhood (50 chromatogram value pairs above and below the respective peak's borders). The peak error is estimated as the difference of the smoothed and the unsmoothed peak. A calculated peak area is accepted in case the calculated peak area is bigger than the estimated error and the peak value is at least twice the estimated background noise, otherwise the peak area is set to zero. The acceptance process is applied in automated peak area determination only. In case of interactive peak determination this process is replaced by the operator's decision. In order to demonstrate the quantification capabilities of MASPECTRAS two samples where mixed at different ratios and quantified with MSQuant [43], PepQuan (provided with the Bioworks browser from SEQUEST), and MASPECTRAS. The results are described in the section "Quantitative analysis", the experiment in the section "Experimental procedures".

#### Visualization tools

**Figure 2:**
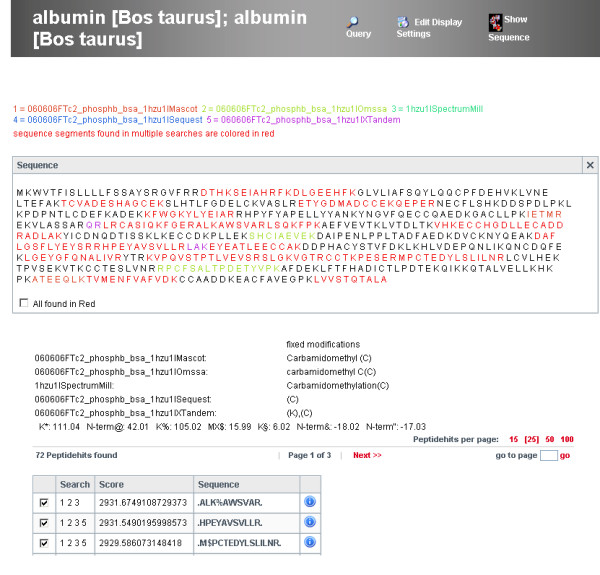
**Combined view of the results from the search engines**. The combined result view shows the comparison of 5 different search engines (SEQUEST, Mascot, Spectrum Mill, X! Tandem, and OMSSA) for bovine serum albumin (see experimental procedures for details). The line on the top lists the search results displayed in color. Sequence segments found only in one of the searches have the corresponding color whereas sequence segments found in multiple searches are colored red. The possible peptide modifications are shown under the protein sequence box. Three types of peptide modifications were defined: ICPL-light (K%), ICPL-heavy (K*), and oxidized methionine (MX$). X! Tandem generates additional modifications at the N-terminus (N-term@, N-term&, and N-term"). X! Tandem does not provide the possibility to search variable modification states on one amino acid. Therefore, for the X! Tandem search a fixed modification at K(+105.02) and a variable modification (K§6.02) has been applied. In the last table the peptides are listed and only one representative for the peptide at this modification state is shown.

MASPECTRAS allows the storage and comparison of search results from the search engines SEQUEST, Mascot, Spectrum Mill, X! Tandem, and OMSSA matched to different sequence databases merged in a single user-definable view (Figure 2). MASPECTRAS provides customizable (clustered) protein, peptide, spectrum, and chromatogram views, as well as a view for the quantitative comparison.

**Figure 3:**
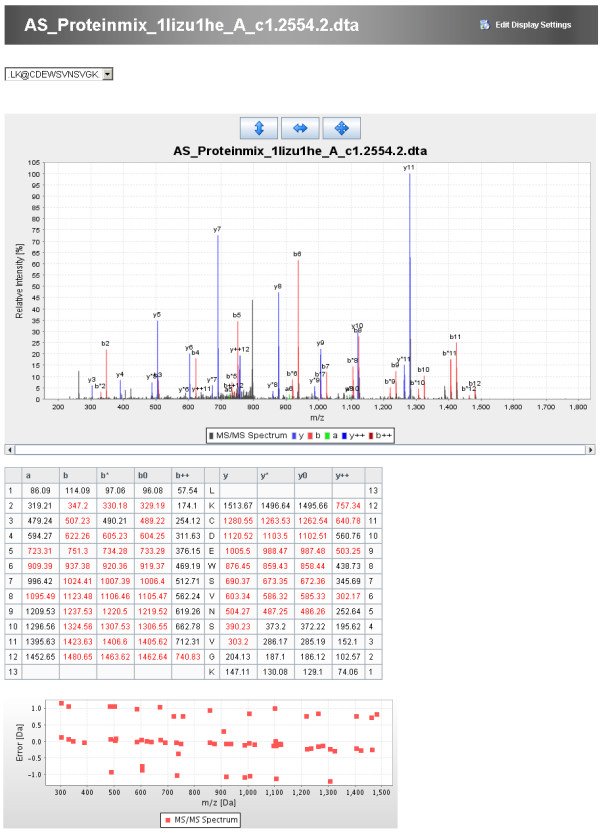
**Spectrum viewer of MASPECTRAS**. The spectrum viewer offers the selection of different ion series, the change to other peptide hits, zooming- and printing possibilities.

**Figure 4:**
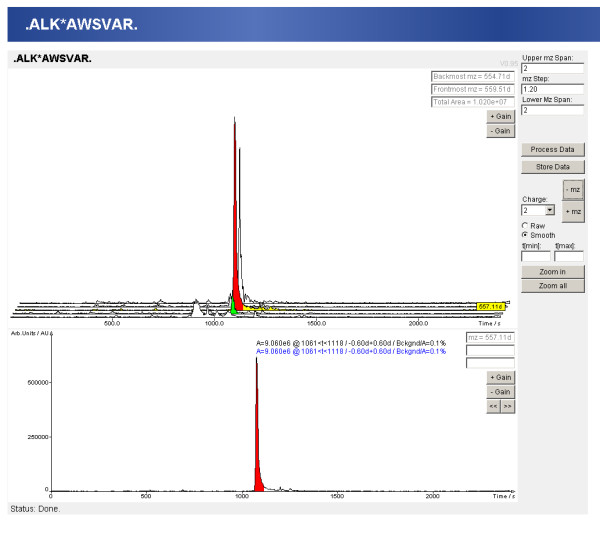
**Chromatogram viewer for the quantification**. The raw data is filtered with the m/z of the peptide found. The calculated chromatogram and the chromatograms of the neighborhood are displayed in the first view. The second view shows the selected chromatogram (the yellow colored one in the first view). Additional peaks can be added and stored peaks (colored red) can be removed. The manually selected peaks are displayed in green. The chromatogram viewer allows changing the m/z step-size, the number of displayed neighborhood chromatograms, and the charge state.

The clustered protein view displays one representative for each protein cluster. In the peptide centric view the peptides with the same modifications are combined together and only the representative with the highest score is displayed. The spectrum viewer of MASPECTRAS enables manual inspection of the data by providing customizable zooming and printing features (Figure 3). The chromatogram viewer allows manual definition of the peak areas (Figure 4). The chromatograms of all charge states of the identified peptide are displayed. The quantitative comparison view offers the possibility to compare peptides with two different post translational modifications (PTMs) or with one PTM and an unmodified version. The calculated peaks are displayed graphically together with a regression line.

#### PRIDE export

MASPECTRAS has been designed to comply with the MIAPE requirements and provide researchers export possibilities to other file formats (Excel, Word, and plain text). Additionally, the export to the PRIDE XML format is possible directly from the protein and peptide views and the resulting file can be submitted to PRIDE repository [7].

### Analysis of large proteomics data set

To demonstrate the utility of the MASPECTRAS we used data from a large-scale study recently published by Kislinger *et al. *[44]. We analyzed the data from the heart cytosol compartment which comprised 84 SEQUEST searches performed against a database obtained from the authors (downloadable at the MASPECTRAS application, see availability and requirements) containing the same amount of "decoy" proteins presented in inverted amino acid orientation. The files were imported, parsed, the data analyzed and the results exported in PRIDE format. In the study of Kislinger *et al. *a protein was accepted with a minimum of two high scoring spectra with a likelihood value >95% (calculated by STATQUEST [45]), which resulted in 698 protein identifications in the cytosol compartment. Applying the same filter criteria and using the PeptideProphet algorithm implemented in MASPECTRAS resulted in 570 protein identifications (81.7%). The results of this analysis are shown in additional file 3 and the data can be downloaded at the MASPECTRAS application (see availability and requirements).

### Quantitative analysis

To evaluate the performance of the quantification tool we initiated a controlled experiment using mixture of ICPL-labeled (Isotope Coded Protein Label) proteins (see experimental procedures). ICPL-labeled probes were mixed at 7 different ratios in triplicates (1:1, 2:1, 5:1 10:1, 1:2, 1:5 and 1:10). To demonstrate the capabilities of MASPECTRAS, the quantitative analysis was performed with MSQuant [43], PepQuan (Bioworks 3.2 – Thermo Electron), and ASAPRatio as implemented in MASPECTRAS. Due to the fact that MSQuant lacks the ability to quantify samples in centroid mode, the automatic quantification of MSQuant and MASPECTRAS has been performed on profile mode data. Additionally we compared the automatic quantification of MASPECTRAS in centroid mode and observed no significant deviation (data not shown).

Since in the centroid mode the data amount is smaller (~1/8) the manual review and correction of the automatically calculated results has been conducted with centroid mode data. The reasons for the manual correction are: (i) there are additional peaks in a chromatogram in the m/z neighborhood; (ii) the found peptides are not in the main peak but in a neighboring smaller peak. A ratio between each found light and heavily labeled peptide has been calculated, and from those ratios the mean value, the standard deviation, the relative error, and a regression line has been calculated as well (with the integrated PTM quantitative comparison tool described in the visualization tools section). A filter for outlier removal has been applied to the automatically calculated ratios. For the manual evaluation, these automatically removed peptides were checked manually and the misquantifications due to the above mentioned reasons could be corrected. Therefore the number of manually accepted peptides could be higher than the automatically accepted ones. The performance of the quantification with ASAPRatio integrated in MASPECTRAS was superior compared to both MSQuant and PepQuan. Furthermore, for all ratios the relative error calculated was considerably lower than the relative error obtained with MSQuant and PepQuan (see table 1 and for more detailed information see additional file 4 for a direct comparison between MSQuant, PepQuan, and MASPECTRAS).

**Table 1:** Summary of quantitative analysis with MASPECTRAS, MSQuant and PepQuan

	10 heavy to 1 light					1 heavy to 10 light			
	
	MSQuant	PepQuan	MASPECTRAS			MSQuant	PepQuan	MASPECTRAS	
	
	auto	manual	auto	manual		auto	manual	auto	manual
	
# peptides	22	33	27	40	# peptides	20	82	28	39
mean	4.64	8.94	8.31	9.85	mean	7.54	7	9.77	9.29
stdev	4.83	4.9	2.85	2.99	stdev	4.94	2.51	3.96	1.92
CV %	104.09%	54.81%	34.30%	30.36%	CV %	65.52%	35.86%	40.53%	20.67%
	
ratio: heavy/light					ratio: light/heavy				
	
	5 heavy to 1 light					1 heavy to 5 light			
	
	MSQuant	PepQuan	MASPECTRAS			MSQuant	PepQuan	MASPECTRAS	
	
	auto	manual	auto	manual		auto	manual	auto	manual
	
# peptides	14	43	50	53	# peptides	16	67	41	40
mean	2.94	4.27	4.16	4.67	mean	13.36	3.74	4.25	4.84
stdev	2.3	1.69	1.56	1.12	stdev	5.18	1.36	1.15	0.93
CV %	78.23%	39.58%	37.50%	23.98%	CV %	38.77%	36.36%	27.06%	19.21%
	
ratio: heavy/light					ratio: light/heavy				
	
	2 heavy to 1 light					1 heavy to 2 light			
	
	MSQuant	PepQuan	MASPECTRAS			MSQuant	PepQuan	MASPECTRAS	
	
	auto	manual	auto	manual		auto	manual	auto	manual
	
# peptides	25	50	48	72	# peptides	16	74	42	47
mean	1.048	2.17	2.07	2.03	mean	4.24	2.07	2.11	1.94
stdev	1.15	0.7	0.71	0.54	stdev	4.97	3.04	0.63	0.3
CV %	109.73%	32.26%	34.30%	26.60%	CV %	117.22%	146.86%	29.86%	15.46%
	
ratio: heavy/light					ratio: light/heavy				
	
	1 heavy to 1 light								
	
	MSQuant	PepQuan	MASPECTRAS						
	
	auto	manual	auto	manual					
	
# peptides	15	67	98	77					
mean	0.92	1.28	0.97	0.99					
stdev	0.46	0.48	0.24	0.19					
CV %	49.30%	37.50%	24.74%	19.10%					

A filter for outlier removal has been applied to the automatically calculated ratios in MASPECTRAS. For the manual evaluation, these automatically removed peptides were checked manually and the misquantification due to wrong peak detection could be corrected. Therefore the amount of manually accepted peptides could be higher than the automatically accepted ones. The quantification with ASAPRatio integrated in MASPECTRAS performed superior compared to both, MSQuant and PepQuan. Furthermore, for all ratios the relative error calculated was considerably lower than the relative error obtained with MSQuant and PepQuan.

## Discussion

We have developed an integrated platform for the analysis and management of proteomics LC-MS/MS data using state-of-the-art software technology. The uniqueness of the platform lies in the MIAPE compliance, PRIDE export, and the scalability of the system for computationally intensive tasks, in combination of common features for data import from common search engines, integration of peptide validation, protein grouping and quantification tools.

MIAPE compliance and PRIDE export are necessary to disseminate data and effectively analyze a proteomics experiment. As more and more researchers are adopting the standards, public repositories will not only enhance data sharing but will also enable data mining within and across experiments. Surprisingly, although standards for data representation have been widely accepted, the necessary software tools are still missing. This can be partly explained by the volume and complexity of the generated data and by the heterogeneity of the used technologies. We have therefore positioned the beginning of the analytical pipeline of MASPECTRAS at the point at which the laboratory workflows converge, i.e. analysis of the data generated by the search engines.

The capability to import and parse data from five search engines makes the platform universal and independent of the workflow performed by the proteomics research group. The system is not confined to a specific manufacturer and can therefore be used in labs equipped with different instruments. Moreover, MASPECTRAS is a system that provides the basis for consensus scoring between MS/MS search algorithms. It was recently suggested that the interpretation of the results from proteomics studies should be based on the analysis of the data using several search engines [21]. Importing and parsing the results from search engines and side-by-side graphical representation of the results is a prerequisite for this type of analysis and would enhance correct identification of peptides. The results of the validation of our system using large proteomics data sets further support this observation. The differences in the results of the analyses are due to the different algorithms used for the likelihood calculation. In our system PeptideProphet [37] was used whereas in the study by Kislinger *et al. *[44] STATQUEST [45] was applied. We have selected PeptideProphet algorithm based on the results of a benchmark study [21] in which PeptideProphet was ranked first with respect to the number of correctly identified peptide spectra. This study by Kapp *et al. *[21] showed also that the concordance between MS/MS search algorithms can vary up to 55% (335 peptides were identified by all four algorithms out of possible 608 hits). Important considerations when carrying out MS/MS database searches are not only the choice of the search engine, but also the selection of search parameters, the search strategy, and the chosen protein sequence database. Evaluation of the performance of the used algorithms was beyond the scope of this study. Further work need to be carried out to determine the number of independent scoring functions necessary to allow automated validation of peptide identifications. It should be noted that inclusion of additional validation algorithms in MASPECTRAS is straightforward due to the flexibility of the platform and the use of standard software technology.

The integration of peptide validation, protein grouping and quantification algorithms in conjunction with visualization tools is important for the usability and acceptability of the system. Particularly the inclusion of a quantification algorithm in the pipeline is of interest since more and more quantitative studies are initiated. We have selected the ASAPRatio algorithm for automated statistical analysis of protein abundance ratios [41] and integrated it into our platform. The results of our validation experiment showed that the performance of ASAPRatio was superior to MSQuant and PepQuan. Again, the modularity of the platform allows future integration of other quantification algorithms. Moreover, the use of three-tier software architecture in which the presentation, the calculation and the database part are separated enables not only easier maintenance but also future changes like inclusion of additional algorithms as well as distribution of the computing load to several servers. We made use of the flexibility of this concept and developed a module for distributing the load to a computing cluster (JClusterService, see implementation). Tests with the ASAPRatio algorithm showed that the computing time decreases linearly with the number of used processors.

## Conclusion

In summary, a comprehensive platform has been developed for the management of proteomics data in a MIAPE compliant manner. MASPECTRAS (i) provides the amenities needed for analysis, (ii) features an automated analysis pipeline and unique analysis tools, (iii) provides an easy export functionality for the submission of the data to public repositories, and (iv)is capable of managing the growing amount of mass spectrometry data in a scalable manner using parallel computing. Given the unique features and the flexibility due to the use of standard software technology, our platform represents significant advance and could be of great interest to the proteomics community.

## Experimental procedures

### Materials

Proteins were purchased from Sigma as lyophylized, dry powder. Solvents (HPLC grade) and chemicals (highest available grade) were purchased from Sigma, TFA (trifluoroacetic acid) was from Pierce. The ICPL (isotope coded protein label) chemicals kit was from Serva Electrophoresis this kit contained reduction solution with TCEP (Tris (2-carboxy-ethyl) phosphine hydrochloride), cysteine blocking solution with IAA (Iodoacetamide), stop solutions I and II and the labeling reagent nicotinic acid N-hydroxysuccinimide ester as light (6 ^12^C in the nicotinic acid) and heavy (6 ^13 ^C) form as solutions. Trypsin was purchased from Sigma at proteomics grade.

### ICPL labeling of proteins

Proteins bovine serum albumin [GenBank:AAA51411.1], human apotransferrin [ref:NP_001054.1] and rabbit phosphorylase b [PDB:8GPB] were dissolved with TEAB (Tetraammoniumbicarbonate) buffer (125 mM, pH 7.8) in three vials to a final concentration of 5 mg/ml each. A 40 μl aliquot was used for reduction of disulfide bonds between cysteine side-chains and blocking of free cysteines. For reduction of disulfide bonds 4 μl of reduction solution were added to the aliquot and the reaction was carried out for 35 min at 60°C. After cooling samples to room temperature, 4 μl of cysteine blocking solution were added and the samples were sat in a dark cupboard for 35 min. To remove excess of blocking reagent 4 μl of stop solution I were added and samples were put on a shaker for 20 minutes. Protein aliquots were split to two samples which contained 20 μl each. First row of samples was labeled with the ^12^C isotope by adding 3 μl of the nicotinic acid solution which contained the light reagent. Second row was labeled with the heavy reagent and labeling reaction was carried out for 2 h and 30 min while shaking at room temperature.

### Proteolytic digest of proteins

Protein solutions were diluted using 50 mM NH_4_HCO_3 _solution to a final volume of 90 μl. 10 μl of a fresh prepared trypsin solution (2.5 μg/μl) were added and the proteolysis was carried out at 37°C over night in an incubator. The reaction was stopped by adding 10μl of 10% TFA. The peptide solutions were diluted with 0.1 % TFA to give 1 nM final concentration. From these stock solutions samples for MS/MS analysis which contained defined ratios of heavy and light were made up by mixing the solutions of light and heavy labeled peptides.

### HPLC and mass spectrometry

To separate peptide mixtures prior to MS analysis, nano reverse phase high-performance liquid chromatography (nanoRP-HPLC) was applied on the Ultimate 2 Dual Gradient HPLC system (Dionex, buffer A: 5% acetonitrile (ACN), 0.1% TFA, buffer B: 80% ACN, 0.1% TFA) on a PepMap separation column (Dionex, C18, 150 mm × 75 μm × 3 μm, 300 A). 500 fM of each mixture was separated three times using the same trapping and separation column to reduce the quantification error which comes from HPLC and mass spectrometry. A gradient from 0% B to 50% B in 48 min was applied for the separation; peptides were detected at 214 and 280 nm in the UV detector. The exit of the HPLC was online coupled to the electrospray source of the LTQ mass spectrometer (Thermo Electron). Samples were analyzed in centroid mode first to test digest and labeling quality. For the quantitative analysis the LTQ was operated in enhanced profile mode for survey scans to gain higher mass accuracy. Samples were mass spectrometrically analyzed using a top one method, in which the most abundant signal of the MS survey scan was fragmented in the subsequent MS/MS event in the ion trap. Although with this method a lower number of MS/MS spectra were acquired, the increased number of MS scans leads to a better determination of the eluting peaks and therefore provides improved quantification of peptides.

Data analysis was done with the Mascot Daemon [27] (Matrix Science), BioWorks 3.2 [26] (Thermo Electron) software packages using an in house database. To demonstrate the merging of results from all of the mentioned search engines the ICPL labeled probes at an ratio of 1:1 were searched with Spectrum Mill A.03.02 (Agilent Technologies) [28], X! Tandem [29] (The Global Proteome Machine Organization) version 2006.04.01, and OMSSA 1.1.0 [30] (NCBI). The results were uploaded to MASPECTRAS and quantified automatically.

## Availability and requirements

• Project name: MASPECTRAS

• Stable instance of MASPECTRAS:  (here datasets for the publication are downloadable)

• Project home page: 

• Operating system: Solaris, Linux, Windows; the JClusterService requires a unix/linux system

• Programming language: Java

• Other requirements: Java JDK 1.5.x, Oracle™ 9i or PostgreSQL™ 8.0.x or MySQL™ 4.1.xx/5.0.xx, server with at least 1 GB of main memory (2 GB are recommended)

• License: IGB-TUG Software License

• Any restrictions to use by non-academics: IGB-TUG Software License needed

Installation: step-by-step instructions are provided at the projects web site together with files and scripts necessary.

The JBoss instance housing the web-interface for the stable instance of MASPECTRAS is currently running on a dual Opteron™ system (Sun™ V20z) under CentOS-Linux 4.5 accessing an Oracle™ 9i database instance on a Sun™ V880 running under Solaris™ 9 as database management system. Additionally the application server is attached to a Storage Area Network (SAN) with a capacity of 7.7 Terabytes. Regarding the high-performance computing infrastructure, MASPECTRAS accesses a 50 CPU computing cluster running under Rocks/CentOS-Linux 4.0 and submitting the calculation tasks via Sun Grid Engine (SGE) to the Intel Xeon based cluster nodes.

## Authors' contributions

JH designed the current version of MASPECTRAS. He was responsible for the implementation of the database, the development the data presentation and many parts of the business logic. GS, AS^1^, TRB and EK implemented most of the parts of the analysis pipeline. GS developed the JClusterService and the services provided for MASPECTRAS. TRB integrated PeptideProphet, AS^1 ^the protein clustering pipeline, and EK the peptide quantification and the chromatogram viewer. RR implemented the PRIDE data export. AS^2 ^and KM conducted the proteomics experiments. JH and AS^2 ^analyzed the biological data. KM and GGT contributed to conception and design. ZT was responsible for the overall conception and project coordination. All authors gave final approval of the version to be published.

## Supplementary Material

Additional file 1 Comparison of MASPECTRAS to other proteomics tools. In this table the features of MASPECTRAS and other proteomics tools are listed next to one another. Click here for file

Additional file 2 MASPECTRAS database schema. The database schema of the MASPECTRAS application. Click here for file

Additional file 3 Evaluation of the heart cytosol data of the study by Kislinger. The data shows the found proteins by the study from Kislinger and the ones by MASPECTRAS. For both of them only proteins with at least 2 first hit high-scoring spectra are accepted. To identify a high-scoring spectrum in the study of Kislinger a likelihood p value < 0.05 of the STATQUEST score has been used, while in MASPECTRAS a PeptideProphet score > 0.95 has been applied. Click here for file

Additional file 4 Quantification results and comparison with MSQuant and PepQuan. This zipped folder contains the comparison of the quantification results from MASPECTRAS, MSQuant, and PepQuan (QuantificationComparisonOfPrograms.xls), the detailed quantification results of MSQuant (MSQuantResults.xls), PepQuan (PepQuanResults.xls), the automatic quantification with MASPECTRAS (ICPL_Protmix_li_he_Automatic.xls), and the manual evaluation using centroid mode data with MASPECTRAS (ICPL_Protmix_li_he_Centroid_Manual.xls). Furthermore the calculated regression lines of MASPECTRAS are included (PNG-files). The original data files used for the quantification are downloadable directly at the MASPECTRAS application (see availability and requirements). Click here for file
