# Supplementary figures and images for: MASPECTRAS: a platform for management and analysis of proteomics LC-MS/MS data
Source: BMC Bioinformatics. 2007 Jun 13;8:197. doi: 10.1186/1471-2105-8-197 (PMC1906842; doi:10.1186/1471-2105-8-197)

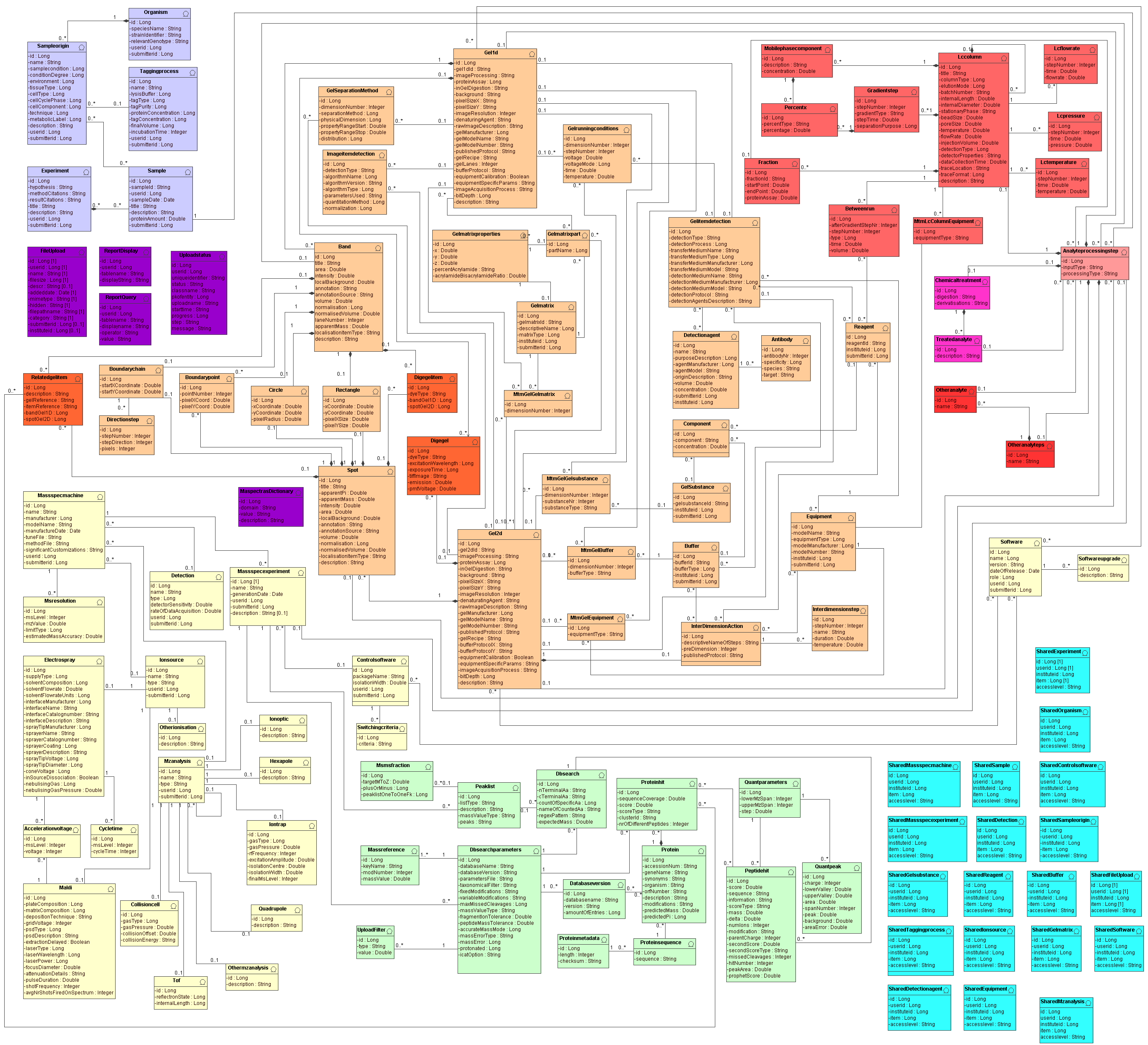

Supplement: Additional file 2 — MASPECTRAS database schema. The database schema of the MASPECTRAS application. [file 1471-2105-8-197-S2.tiff]

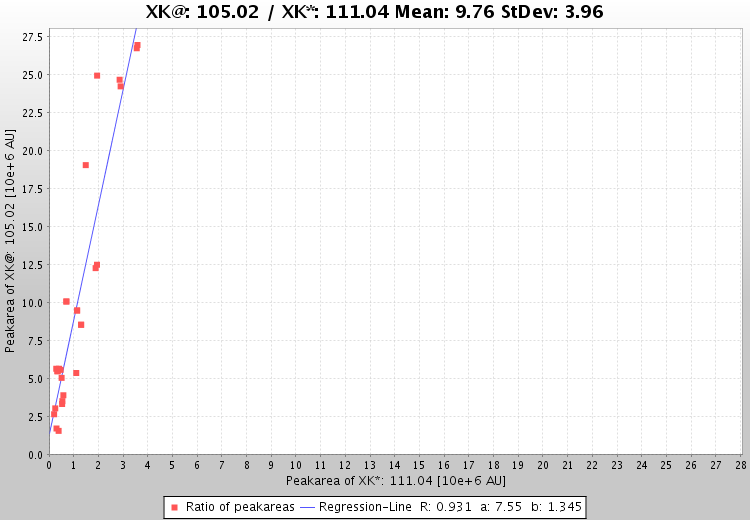

Supplement: Additional file 4 — Quantification results and comparison with MSQuant and PepQuan. This zipped folder contains the comparison of the quantification results from MASPECTRAS, MSQuant, and PepQuan (QuantificationComparisonOfPrograms.xls), the detailed quantification results of MSQuant (MSQuantResults.xls), PepQuan (PepQuanResults.xls), the automatic quantification with MASPECTRAS (ICPL_Protmix_li_he_Automatic.xls), and the manual evaluation using centroid mode data with MASPECTRAS (ICPL_Protmix_li_he_Centroid_Manual.xls). Furthermore the calculated regression lines of MASPECTRAS are included (PNG-files). The original data files used for the quantification are downloadable directly at the MASPECTRAS application (see availability and requirements). [file 1471-2105-8-197-S4.zip › addData4/ICPL_Protmix_10li_1he_Automatic.png]

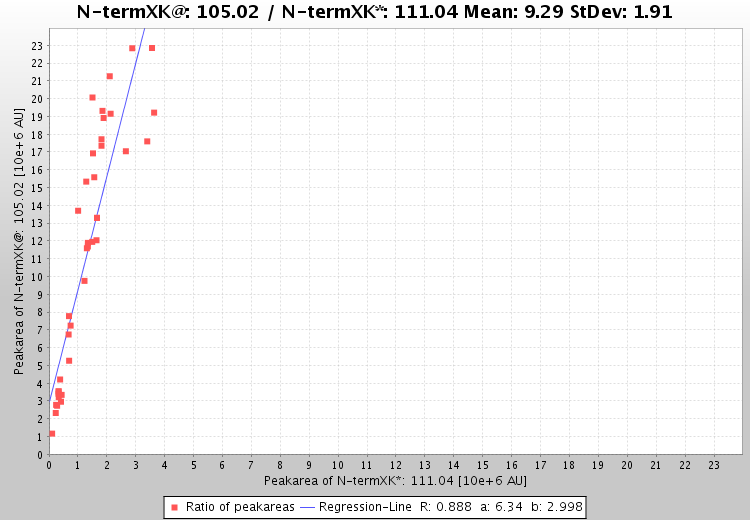

Supplement: Additional file 4 — Quantification results and comparison with MSQuant and PepQuan. This zipped folder contains the comparison of the quantification results from MASPECTRAS, MSQuant, and PepQuan (QuantificationComparisonOfPrograms.xls), the detailed quantification results of MSQuant (MSQuantResults.xls), PepQuan (PepQuanResults.xls), the automatic quantification with MASPECTRAS (ICPL_Protmix_li_he_Automatic.xls), and the manual evaluation using centroid mode data with MASPECTRAS (ICPL_Protmix_li_he_Centroid_Manual.xls). Furthermore the calculated regression lines of MASPECTRAS are included (PNG-files). The original data files used for the quantification are downloadable directly at the MASPECTRAS application (see availability and requirements). [file 1471-2105-8-197-S4.zip › addData4/ICPL_Protmix_10li_1he_Centroid_Manual.png]

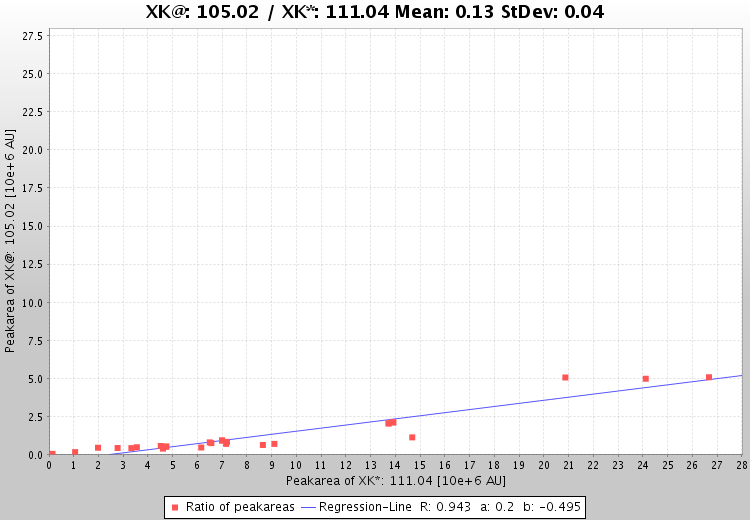

Supplement: Additional file 4 — Quantification results and comparison with MSQuant and PepQuan. This zipped folder contains the comparison of the quantification results from MASPECTRAS, MSQuant, and PepQuan (QuantificationComparisonOfPrograms.xls), the detailed quantification results of MSQuant (MSQuantResults.xls), PepQuan (PepQuanResults.xls), the automatic quantification with MASPECTRAS (ICPL_Protmix_li_he_Automatic.xls), and the manual evaluation using centroid mode data with MASPECTRAS (ICPL_Protmix_li_he_Centroid_Manual.xls). Furthermore the calculated regression lines of MASPECTRAS are included (PNG-files). The original data files used for the quantification are downloadable directly at the MASPECTRAS application (see availability and requirements). [file 1471-2105-8-197-S4.zip › addData4/ICPL_Protmix_1li_10he_Automatic.png]

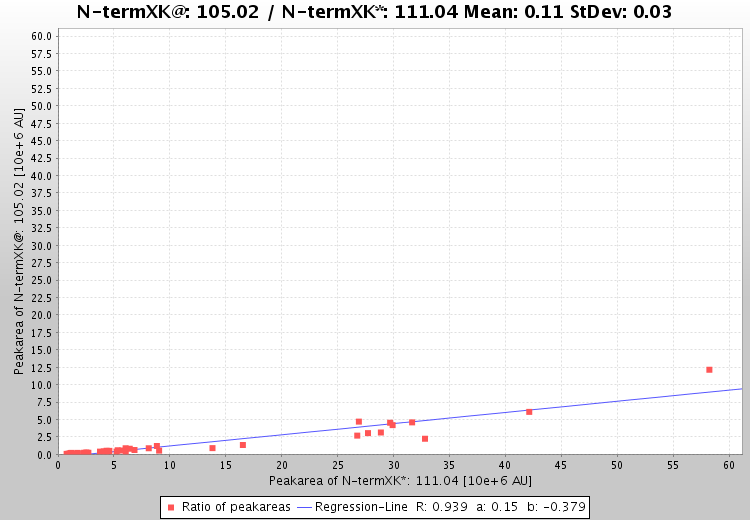

Supplement: Additional file 4 — Quantification results and comparison with MSQuant and PepQuan. This zipped folder contains the comparison of the quantification results from MASPECTRAS, MSQuant, and PepQuan (QuantificationComparisonOfPrograms.xls), the detailed quantification results of MSQuant (MSQuantResults.xls), PepQuan (PepQuanResults.xls), the automatic quantification with MASPECTRAS (ICPL_Protmix_li_he_Automatic.xls), and the manual evaluation using centroid mode data with MASPECTRAS (ICPL_Protmix_li_he_Centroid_Manual.xls). Furthermore the calculated regression lines of MASPECTRAS are included (PNG-files). The original data files used for the quantification are downloadable directly at the MASPECTRAS application (see availability and requirements). [file 1471-2105-8-197-S4.zip › addData4/ICPL_Protmix_1li_10he_Centroid_Manual.png]

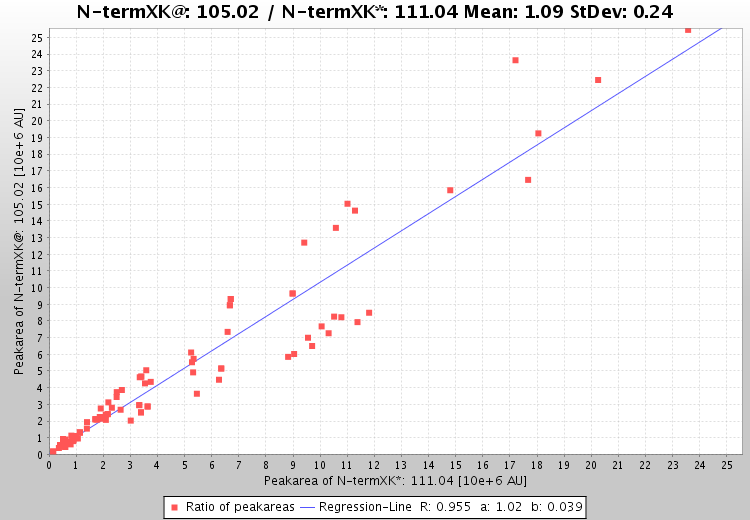

Supplement: Additional file 4 — Quantification results and comparison with MSQuant and PepQuan. This zipped folder contains the comparison of the quantification results from MASPECTRAS, MSQuant, and PepQuan (QuantificationComparisonOfPrograms.xls), the detailed quantification results of MSQuant (MSQuantResults.xls), PepQuan (PepQuanResults.xls), the automatic quantification with MASPECTRAS (ICPL_Protmix_li_he_Automatic.xls), and the manual evaluation using centroid mode data with MASPECTRAS (ICPL_Protmix_li_he_Centroid_Manual.xls). Furthermore the calculated regression lines of MASPECTRAS are included (PNG-files). The original data files used for the quantification are downloadable directly at the MASPECTRAS application (see availability and requirements). [file 1471-2105-8-197-S4.zip › addData4/ICPL_Protmix_1li_1he_Automatic.png]

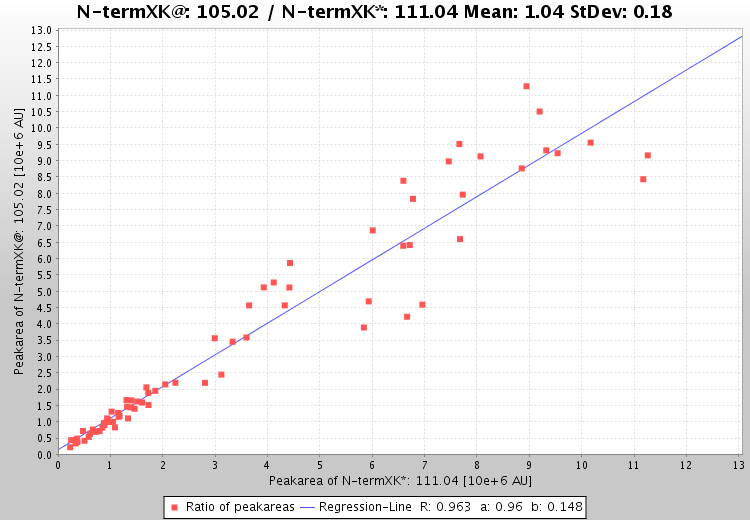

Supplement: Additional file 4 — Quantification results and comparison with MSQuant and PepQuan. This zipped folder contains the comparison of the quantification results from MASPECTRAS, MSQuant, and PepQuan (QuantificationComparisonOfPrograms.xls), the detailed quantification results of MSQuant (MSQuantResults.xls), PepQuan (PepQuanResults.xls), the automatic quantification with MASPECTRAS (ICPL_Protmix_li_he_Automatic.xls), and the manual evaluation using centroid mode data with MASPECTRAS (ICPL_Protmix_li_he_Centroid_Manual.xls). Furthermore the calculated regression lines of MASPECTRAS are included (PNG-files). The original data files used for the quantification are downloadable directly at the MASPECTRAS application (see availability and requirements). [file 1471-2105-8-197-S4.zip › addData4/ICPL_Protmix_1li_1he_Centroid_Manual.png]

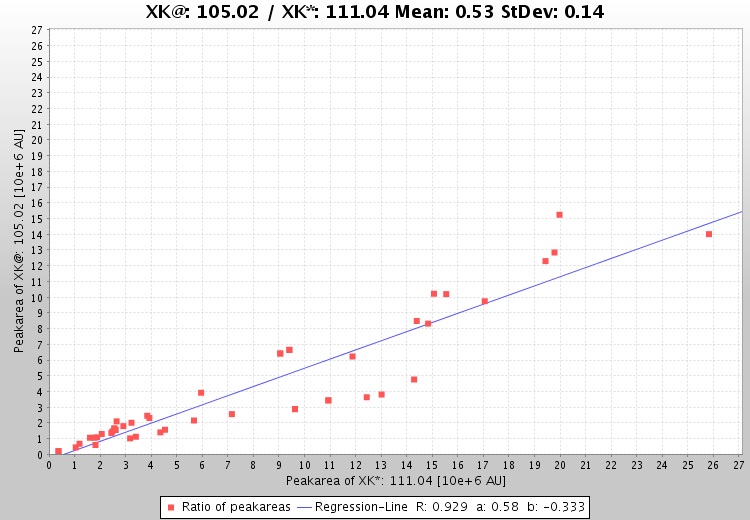

Supplement: Additional file 4 — Quantification results and comparison with MSQuant and PepQuan. This zipped folder contains the comparison of the quantification results from MASPECTRAS, MSQuant, and PepQuan (QuantificationComparisonOfPrograms.xls), the detailed quantification results of MSQuant (MSQuantResults.xls), PepQuan (PepQuanResults.xls), the automatic quantification with MASPECTRAS (ICPL_Protmix_li_he_Automatic.xls), and the manual evaluation using centroid mode data with MASPECTRAS (ICPL_Protmix_li_he_Centroid_Manual.xls). Furthermore the calculated regression lines of MASPECTRAS are included (PNG-files). The original data files used for the quantification are downloadable directly at the MASPECTRAS application (see availability and requirements). [file 1471-2105-8-197-S4.zip › addData4/ICPL_Protmix_1li_2he_Automatic.png]

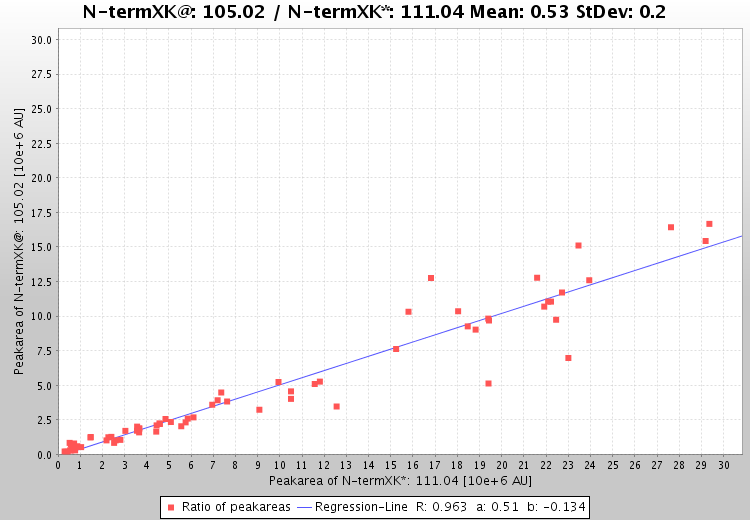

Supplement: Additional file 4 — Quantification results and comparison with MSQuant and PepQuan. This zipped folder contains the comparison of the quantification results from MASPECTRAS, MSQuant, and PepQuan (QuantificationComparisonOfPrograms.xls), the detailed quantification results of MSQuant (MSQuantResults.xls), PepQuan (PepQuanResults.xls), the automatic quantification with MASPECTRAS (ICPL_Protmix_li_he_Automatic.xls), and the manual evaluation using centroid mode data with MASPECTRAS (ICPL_Protmix_li_he_Centroid_Manual.xls). Furthermore the calculated regression lines of MASPECTRAS are included (PNG-files). The original data files used for the quantification are downloadable directly at the MASPECTRAS application (see availability and requirements). [file 1471-2105-8-197-S4.zip › addData4/ICPL_Protmix_1li_2he_Centroid_Manual.png]

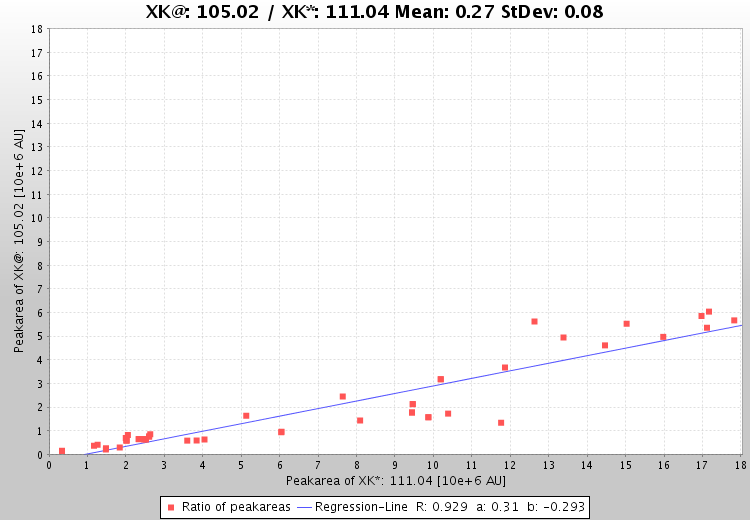

Supplement: Additional file 4 — Quantification results and comparison with MSQuant and PepQuan. This zipped folder contains the comparison of the quantification results from MASPECTRAS, MSQuant, and PepQuan (QuantificationComparisonOfPrograms.xls), the detailed quantification results of MSQuant (MSQuantResults.xls), PepQuan (PepQuanResults.xls), the automatic quantification with MASPECTRAS (ICPL_Protmix_li_he_Automatic.xls), and the manual evaluation using centroid mode data with MASPECTRAS (ICPL_Protmix_li_he_Centroid_Manual.xls). Furthermore the calculated regression lines of MASPECTRAS are included (PNG-files). The original data files used for the quantification are downloadable directly at the MASPECTRAS application (see availability and requirements). [file 1471-2105-8-197-S4.zip › addData4/ICPL_Protmix_1li_5he_Automatic.png]

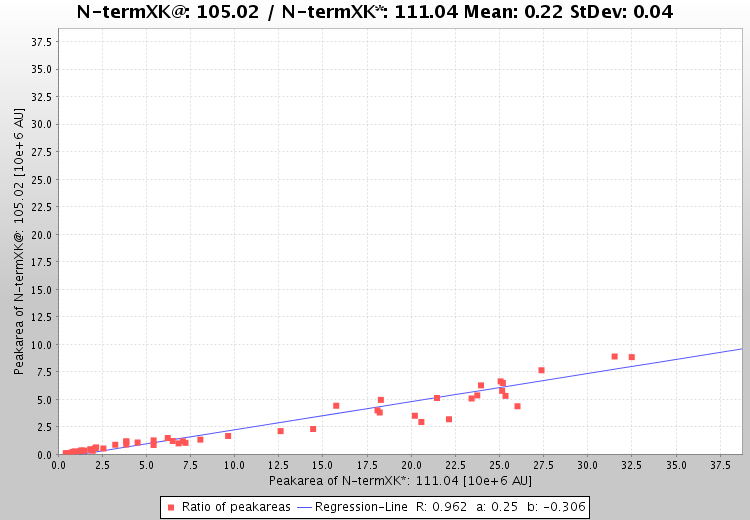

Supplement: Additional file 4 — Quantification results and comparison with MSQuant and PepQuan. This zipped folder contains the comparison of the quantification results from MASPECTRAS, MSQuant, and PepQuan (QuantificationComparisonOfPrograms.xls), the detailed quantification results of MSQuant (MSQuantResults.xls), PepQuan (PepQuanResults.xls), the automatic quantification with MASPECTRAS (ICPL_Protmix_li_he_Automatic.xls), and the manual evaluation using centroid mode data with MASPECTRAS (ICPL_Protmix_li_he_Centroid_Manual.xls). Furthermore the calculated regression lines of MASPECTRAS are included (PNG-files). The original data files used for the quantification are downloadable directly at the MASPECTRAS application (see availability and requirements). [file 1471-2105-8-197-S4.zip › addData4/ICPL_Protmix_1li_5he_Centroid_Manual.png]

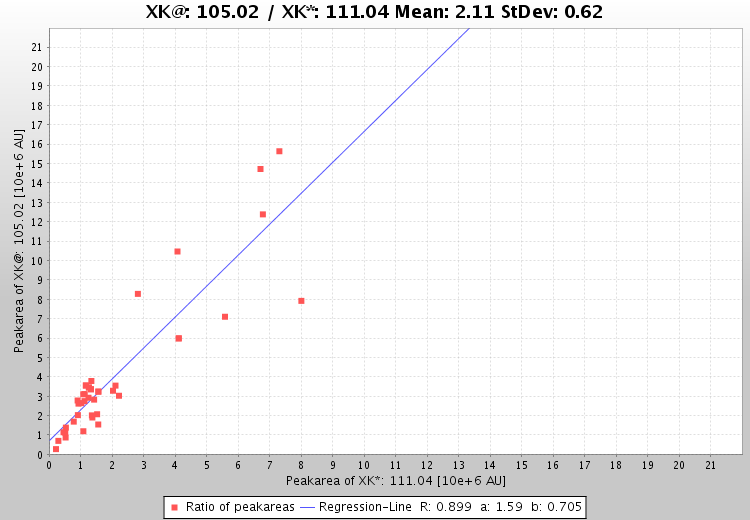

Supplement: Additional file 4 — Quantification results and comparison with MSQuant and PepQuan. This zipped folder contains the comparison of the quantification results from MASPECTRAS, MSQuant, and PepQuan (QuantificationComparisonOfPrograms.xls), the detailed quantification results of MSQuant (MSQuantResults.xls), PepQuan (PepQuanResults.xls), the automatic quantification with MASPECTRAS (ICPL_Protmix_li_he_Automatic.xls), and the manual evaluation using centroid mode data with MASPECTRAS (ICPL_Protmix_li_he_Centroid_Manual.xls). Furthermore the calculated regression lines of MASPECTRAS are included (PNG-files). The original data files used for the quantification are downloadable directly at the MASPECTRAS application (see availability and requirements). [file 1471-2105-8-197-S4.zip › addData4/ICPL_Protmix_2li_1he_Automatic.png]

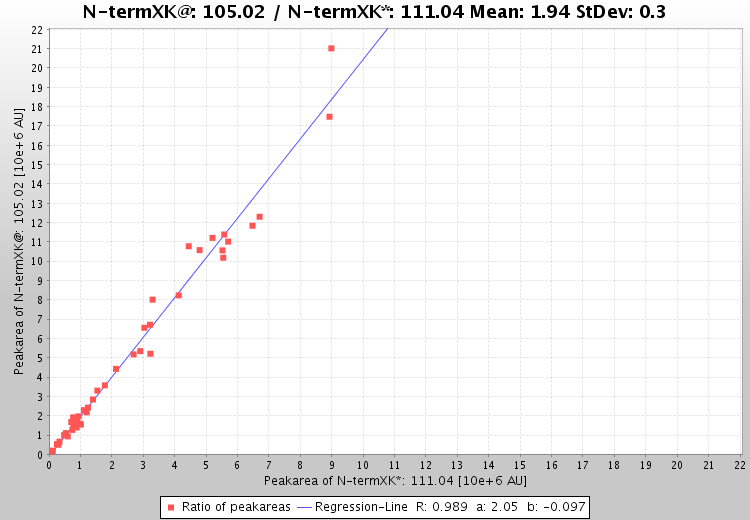

Supplement: Additional file 4 — Quantification results and comparison with MSQuant and PepQuan. This zipped folder contains the comparison of the quantification results from MASPECTRAS, MSQuant, and PepQuan (QuantificationComparisonOfPrograms.xls), the detailed quantification results of MSQuant (MSQuantResults.xls), PepQuan (PepQuanResults.xls), the automatic quantification with MASPECTRAS (ICPL_Protmix_li_he_Automatic.xls), and the manual evaluation using centroid mode data with MASPECTRAS (ICPL_Protmix_li_he_Centroid_Manual.xls). Furthermore the calculated regression lines of MASPECTRAS are included (PNG-files). The original data files used for the quantification are downloadable directly at the MASPECTRAS application (see availability and requirements). [file 1471-2105-8-197-S4.zip › addData4/ICPL_Protmix_2li_1he_Centroid_Manual.png]

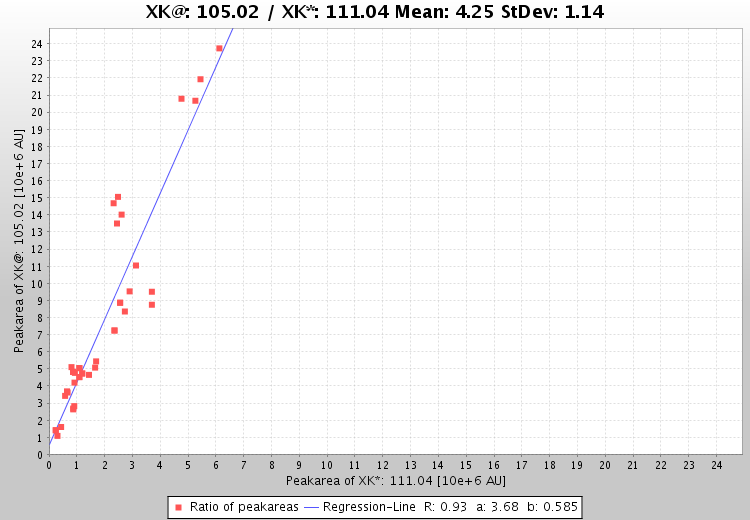

Supplement: Additional file 4 — Quantification results and comparison with MSQuant and PepQuan. This zipped folder contains the comparison of the quantification results from MASPECTRAS, MSQuant, and PepQuan (QuantificationComparisonOfPrograms.xls), the detailed quantification results of MSQuant (MSQuantResults.xls), PepQuan (PepQuanResults.xls), the automatic quantification with MASPECTRAS (ICPL_Protmix_li_he_Automatic.xls), and the manual evaluation using centroid mode data with MASPECTRAS (ICPL_Protmix_li_he_Centroid_Manual.xls). Furthermore the calculated regression lines of MASPECTRAS are included (PNG-files). The original data files used for the quantification are downloadable directly at the MASPECTRAS application (see availability and requirements). [file 1471-2105-8-197-S4.zip › addData4/ICPL_Protmix_5li_1he_Automatic.png]

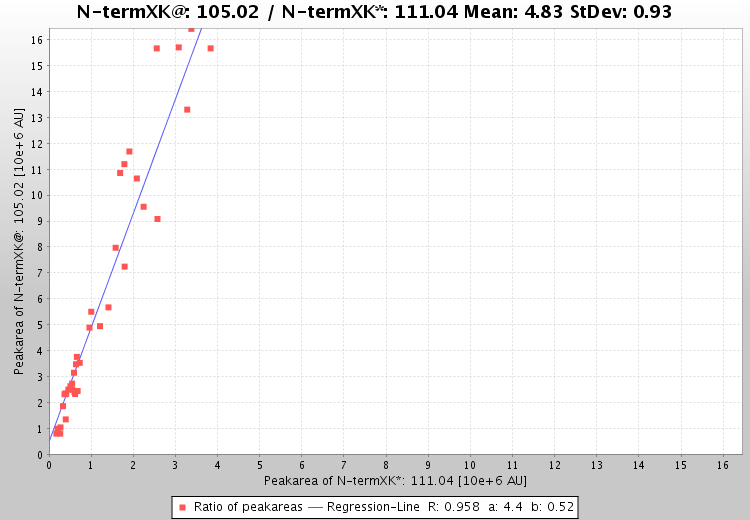

Supplement: Additional file 4 — Quantification results and comparison with MSQuant and PepQuan. This zipped folder contains the comparison of the quantification results from MASPECTRAS, MSQuant, and PepQuan (QuantificationComparisonOfPrograms.xls), the detailed quantification results of MSQuant (MSQuantResults.xls), PepQuan (PepQuanResults.xls), the automatic quantification with MASPECTRAS (ICPL_Protmix_li_he_Automatic.xls), and the manual evaluation using centroid mode data with MASPECTRAS (ICPL_Protmix_li_he_Centroid_Manual.xls). Furthermore the calculated regression lines of MASPECTRAS are included (PNG-files). The original data files used for the quantification are downloadable directly at the MASPECTRAS application (see availability and requirements). [file 1471-2105-8-197-S4.zip › addData4/ICPL_Protmix_5li_1he_Centroid_Manual.png]
